# Unliganded Thyroid Hormone Receptor α Regulates Developmental Timing via Gene Repression in *Xenopus tropicalis*

**DOI:** 10.1210/en.2014-1554

**Published:** 2014-12-02

**Authors:** Jinyoung Choi, Ken-ichi T. Suzuki, Tetsushi Sakuma, Leena Shewade, Takashi Yamamoto, Daniel R. Buchholz

**Affiliations:** Department of Biological Sciences (J.C., L.S., D.R.B.), University of Cincinnati, Cincinnati, Ohio 45221; and Department of Mathematical and Life Sciences (K.T.S., T.S., T.Y.), Graduate School of Science, Hiroshima University, Hiroshima 739-8526, Japan

## Abstract

Thyroid hormone (TH) receptor (TR) expression begins early in development in all vertebrates when circulating TH levels are absent or minimal, yet few developmental roles for unliganded TRs have been established. Unliganded TRs are expected to repress TH-response genes, increase tissue responsivity to TH, and regulate the timing of developmental events. Here we examined the role of unliganded TRα in gene repression and development in *Xenopus tropicalis*. We used transcription activator-like effector nuclease gene disruption technology to generate founder animals with mutations in the *TR*α gene and bred them to produce F1 offspring with a normal phenotype and a mutant phenotype, characterized by precocious hind limb development. Offspring with a normal phenotype had zero or one disrupted *TR*α alleles, and tadpoles with the mutant hind limb phenotype had two truncated *TR*α alleles with frame shift mutations between the two zinc fingers followed by 40–50 mutant amino acids and then an out-of-frame stop codon. We examined TH-response gene expression and early larval development with and without exogenous TH in F1 offspring. As hypothesized, mutant phenotype tadpoles had increased expression of TH-response genes in the absence of TH and impaired induction of these same genes after exogenous TH treatment, compared with normal phenotype animals. Also, mutant hind limb phenotype animals had reduced hind limb and gill responsivity to exogenous TH. Similar results in methimazole-treated tadpoles showed that increased TH-response gene expression and precocious development were not due to early production of TH. These results indicate that unliganded TRα delays developmental progression by repressing TH-response genes.

“The function of apoTR [unliganded thyroid hormone receptor] could be important in premetamorphic amphibians, and it would be particularly interesting to analyze the effect of TR gene inactivation in Xenopus to determine whether TRα has a function as a 'safety lock' to metamorphosis, in which case one would expect *TR*α gene knockout to result in precocious metamorphosis” Chassande (2003) (1).

Few activities of unliganded thyroid hormone receptors (TRs) in regulating developmental events have been established. Unliganded TRs may merely serve as competence factors enabling tissues to respond when the thyroid hormone (TH) is released into circulation later in development. However, unliganded receptors may also play a more active role in regulating developmental events by virtue of gene repression activity in the absence of TH (2). The lethal effect of paired box transcription factor 8^−/−^ mutation (lack of a thyroid gland and TH) compared with the nonlethal effect of *TR*α/*TR*β null mutations in mice points to the developmental actions of TR isoforms in the absence of TH (3, 4). Three specific examples of TRα action in the absence of TH in cerebellum (5), heart (6), and cochlea (7) development have been identified using TRα-deficient mice. These studies showed evidence for TRα-mediated repression of neuron migration or ion channel expression before TH availability, although altered organ function was not observed for cochlea under euthyroid conditions. The paucity of phenotypes attributable to unliganded TR may be due to the presence of low levels of TH from maternal or fetal sources that may conceal potential effects of unliganded TR. Deleterious effects of unliganded TR may be uncovered in pathological hypothyroid conditions, as shown for the cochlea. A useful model to reveal potential effects of unliganded TR in development in both euthyroid and hypothyroid conditions is frog metamorphosis because of the dramatic and well-studied dependence of developmental events on TH and importantly because maternal sources of TH are not present in premetamorphic tadpoles prior to endogenous TH production (8).

First proposed when frog TRs were cloned and then elaborated later (8–10), a dual function model for the role of TR in postembryonic development states the following: 1) TRs act to repress genes involved in developmental progression in the absence of TH to allow larval growth, and 2) upon TH release into circulation, TRs bind TH and induce the previously repressed genes to initiate metamorphosis. Strong evidence exists for the second part of this model, but unequivocal support for the role of unliganded TR in gene regulation and metamorphosis has been lacking.

The dual function model emerged from knowledge of the ligand-dependent action of TRs and the developmental profiles of TR expression and circulating TH levels (10). As in other vertebrates, two gene loci for TR, *TR*α and *TR*β, are found in frogs (11). TRs are nuclear receptors that mediate the TH signal by regulating expression of TH-response genes, depending on the presence or absence of hormone (12, 13). In the absence of TH, TRs bind promoter or enhancer regions of regulated genes and recruit corepressors, nuclear receptor corepressor (NCoR) and silencing mediator of retinoid and thyroid receptors, which deacetylate histones and repress gene expression, and in the presence of TH, a conformational shift in TR favors binding coactivators, including as CBP/p300, coactivator-associated arginine methyltransferase 1, protein arginine methyltransferase, and steroid receptor coactivators, leading to histone acetylation and methylation, altered chromatin structure, and gene induction (8, 10, 14, 15). One or both TR isoforms are expressed in virtually all tissues in the tadpole (16), and TRα expression in particular begins very early in development before the start of feeding (17). TRβ expression levels are very low in premetamorphosis and are highly induced by TH (18). Detectable TH in circulation is coincident with the initiation of metamorphosis and reaches a peak at metamorphic climax (19). Importantly, TH is not detected in circulation until 3 weeks after feeding begins, indicating a substantial amount of time that TRs exist in the unliganded condition.

Extensive indirect mechanistic evidence supports widespread effects of unliganded TR in the premetamorphic tadpole. The initiation of expression of TR correlates with the decreased expression in the TH-direct response genes stromelysin-3 (*ST3*) (20) and sonic hedgehog (21). Also, overexpression of TR/RXR in embryos by mRNA injection precociously repressed these genes to some extent (22). Quantitative chromatin immunoprecipitation analysis in vivo showed that high TR binding to the *TR*β promoter is constitutive (23, 24), and NCoR is recruited there in a TH-dependent manner (25, 26). In functional studies involving NCoR, tail muscle injection of a dominant negative NCoR that blocks endogenous NCoR from binding nuclear receptors increased transcription from coinjected TRα and a reporter plasmid (TRE-tk-luciferase) (26). Also, transgenic overexpression of a dominant-negative NCoR significantly accelerated metamorphic progression (27). On the other hand, TR binding to the TH/basic leucine zipper domain (bZIP) promoter is low or not detectable in the absence of T_3_ (23, 24), yet NCoR is recruited to TH/bZIP in premetamorphosis, even though TR is not well bound (26). Also, in the transgenic overexpression of a dominant negative NCoR, TRβ and TH/bZIP showed no significant derepression, and ST3 and sonic hedgehog showed derepression but not at all time points (27). Thus, available evidence for a role for unliganded TRs in premetamorphosis is based on expression correlations, nonendogenous conditions, and results with NCoR that may not be due to direct interactions with TR. Nevertheless, a role for unliganded TR has not been ruled out. Direct experimental evidence in vivo on endogenous gene regulation and development is required to unequivocally demonstrate a role for unliganded receptor in frogs and reveal potential developmental pathologies in the hypothyroid condition.

In addition to the repression of metamorphic genes and tissue competence, the premetamorphic TR may also contribute to tissue sensitivity/responsivity. Sensitivity refers to a tissue's competence to respond to a given TH concentration, and responsivity refers to a tissue's degree or magnitude of the effect of TH on the tissue at a given TH concentration and time point. Sensitivity and responsivity can be interrelated because of potential similar underlying mechanisms, eg, the expression level of TR. Indeed, TR levels correlate with sensitivity/responsivity to TH among larval tissues (28). Furthermore, the overexpression of TR increases cell sensitivity and responsivity to TH (29, 30). However, the experimental reduction of tissue sensitivity by the reduction of TR expression levels has not previously been accomplished to determine the requirement for TRα in tissue sensitivity or responsivity. Here, as presaged previously in the quote above, we made use of the recently developed gene disruption technology, transcription activator-like effector nucleases (TALENs) (31), to disrupt *TR*α and provide direct and dramatic evidence of the role of unliganded TRα in tissue responsivity, gene repression, and developmental timing.

## Materials and Methods

### TRα TALENs and mRNA synthesis

TALEN plasmids were assembled using the Platinum Gate TALEN kit (Addgene; catalog number 1000000043) as described previously (32). The ptCMV-153/47-VR-HD and ptCMV-153/47-VR-NG vectors were used as destination vectors for the left and right TALENs, respectively. Activity of constructed TALEN plasmids were validated by the human cell-based single-strand annealing assay as previously described (33). TALEN mRNAs were synthesized from plasmid DNA encoding TRα TALENs (right and left arms) linearized with *Xma*I followed by lithium chloride precipitation using T7 mMESSAGE mMACHINE (Life Technologies). To monitor embryo injections, mCherry mRNA from *Kpn*I-linearized CS108-mCherry (a gift from Dr M. Khokha) was also prepared with T7 and lithium chloride precipitation. A mixture of TRα TALEN mRNAs (400 pg for the left arm, 400 pg or the right arm), and mCherry mRNAs (25 pg) were injected into each embryo.

### Animals and microinjection

To induce breeding and obtain offspring, female and male *Xenopus tropicalis* from the laboratory colony were primed with 20 U of human chorionic gonadotropin (Sigma-Aldrich) 14–16 hours before being boosted with 200 U. Freshly fertilized eggs from mated breeding adults in reconstituted reverse osmosis water were collected and dejellied for 5–10 minutes in 3% L-cysteine (Sigma-Aldrich) in 0.1× modified Barth's solution (MBS) (34) and transferred to 3% Ficoll in 0.1× MBS. Dejellied embryos were injected with mRNA into one cell at the two-cell stage. After 3–5 hours, the surviving embryos were transferred to 0.01× MBS. After 2 days, tadpoles hatched and were sorted under a fluorescence dissecting microscope into left side and right side groups based on mCherry fluorescence. Hatched tadpoles were fed finely ground frog brittle (Nasco) twice daily and reared at 26°C with daily water changes. Tadpoles were staged according to Nieuwkoop and Faber (NF) (35). The sex of the tadpoles used is unknown because gonad differentiation begins at NF stage 54 in *Xenopus*, prior to most of our experiments. Some F1 offspring were reared in 0, 2, or 10 nM T_3_ (Sigma) without feeding or 1 mM methimazole (Sigma) with feeding, all with daily water changes. Fluorescence with an RFP filter set and bright-field images were taken using a Leica MZ 16F fluorescence dissection microscope and a Leica DFC420 digital camera. The use of animals in the study was approved by the University of Cincinnati Institutional Animal Care and Use Committee (protocol number 06-10-03-01).

### Genetic analysis

Genomic DNA was prepared from the whole body or tail tip using Quick-gDNA Miniprep (Zymo Research). Up to 500 ng of genomic DNA was used as template for PCR to amplify the region surrounding the TRα TALEN target site (Takara Taq; Takara Bio Inc) that contained 0.2 μM of forward (5′-GGTTTCTTYCGCCGCACCA) (Y stands for C or T) and reverse (5′-ATCCATTGCCATGCCAACGG) primers with the following reaction conditions: 94°C for 5 minutes, 33 cycles at 94°C for 30 seconds, 55°C for 30 seconds, and 72°C for 30 seconds. PCR products were spin column purified (Qiagen), cloned into TOPO T vectors (Invitrogen), and sequenced.

### Quantitative real-time PCR (qPCR)

Tadpole snout-to-vent length, hind limb length, and NF stage of hind limb were measured, and the whole body was snap frozen and stored at −80°C until RNA extraction (TriZol; Invitrogen). One microgram of total RNA was used for cDNA synthesis (high-capacity cDNA reverse transcriptase kit; Applied Biosystems), and one microliter of cDNA was used for each 20-μL qPCR (TaqMan 2× universal PCR master mix; Applied Biosystems) with a qPCR primer/probe set (Applied Biosystems) on a 7300 Real Time PCR system with default reaction conditions (50°C for 2 min and 95°C for 10 min and then 40 cycles of 95°C for 10 sec and 60°C for 1 min) (Applied Biosystems). Primer/probe sets designed to span a large intron were used for ST3 (forward: 5′-GTCAACCAGGTGGAAAATGAGAGTA, reverse: 5′-CACGAATTGTAGACACTGCATCAAA, probe: 5′-CATGCATCAGGCTCTGC), Krüppel-like factor 9 (*KLF9*) (forward: 5′-CCTTAAAGCCCATTACAGAGTCCAT, reverse: 5′-GCAGTCAGGCCACGTACA, probe: 5′-ACAGGTGAACGCCCTTTT), TRβ (forward: 5′-CAAGAGTTGTTGATTTTGCCAAAAA, reverse: 5′-ACATGATCTCCATACAACAGCCTTT, probe: 5′-CTGCCATGTGAAGACC), TRα (forward: 5′-CCACTGGAAACAGCGTAGGA, reverse: 5′-CATGGGAGACTGCCCGATAT, probe: 5′-CTTCCGGCAGAAACT), and rpL8 (forward: 5′-CACAATCCTGAAACCAAGAAAACCA, reverse: 5′-CCACACCACGGACACGT, probe: 5′-AAGGCCAAGAGAAACT). Two technical replicates were used per sample or no-template control. The ddCt method (36) was used to analyze the qPCR results. A one-way ANOVA was performed to test for significant differences among treatments and/or genotypes (JMP statistical software; SAS Institute).

## Results

### TALEN design and construction

**Figure 1.**
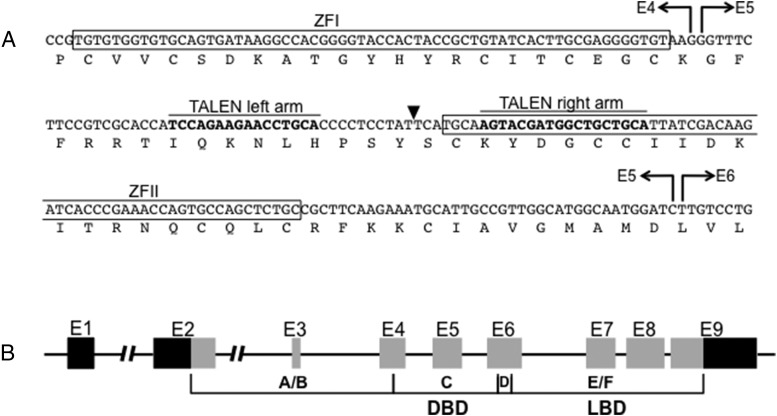
Diagram of TRα TALEN design. A, Binding regions for left and right arms of TRα TALEN (bold nucleotides) flank a spacer region in which a double-stranded break (black triangle) catalyzed by FokI nuclease domains is repaired by nonhomologous end joining. Zinc finger domains, amino acids, and exon boundaries (E4, E5, E6) are indicated. B, *TR*α is composed of nine exons (E1–9), and the targeted region is within exon 5 (E5), part of the coding region for the DNA binding domain (DBD). Black bars in E1 and E9 represent 5′ and 3′ untranslated regions, respectively. A/B, C, D, E/F, nuclear receptor protein domains, LBD, ligand binding domain.

To create TRα knockout frogs, TALENs were designed to target the DNA binding domain of the *TR*α gene of *X. tropicalis* (Figure 1). The TALEN target site is located in between two zinc finger domains followed by the D domain (hinge region) and the E/F domain (ligand binding domain) of TRα (37, 38). The single-strand annealing assay was carried out to evaluate TALEN targeting efficiency, and a TRα TALEN set was confirmed to have high disruption activity (data not shown). TRα TALEN mRNA was injected into fertilized eggs and showed a high survival rate.

### Phenotypes in F0 TRα mutant animals

**Figure 2.**
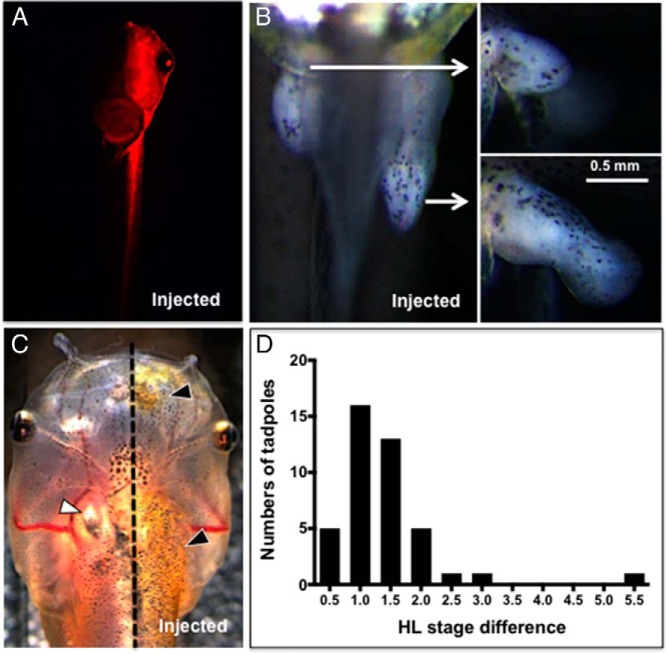
TRα TALEN founder developmental phenotypes. A, mCherry mRNA was coinjected with TRα TALEN mRNA into a single blastomere of two-cell stage embryos. Ventral view of the injected tadpole shows mCherry expression only on the left side of its body. B and C, Hind limb and skin phenotype are shown. B, Ventral view of the lower abdomen and proximal part of tail shows different developmental progression in the hind limbs between uninjected and injected sides. White arrows point to side views of the hind limb on each side to show the alteration of not just limb length but stage of limb development. C, Skin on the injected side of a NF stage 57 tadpole shows advanced iridophore development (gold skin color, black triangles). Thicker skin obscures the ultimobranchial body visible only on the uninjected side (white triangle). The dotted line delineates the uninjected and injected sides. D, Histogram of hind limb stage difference in TRα TALEN founders. When the stage of hind limb development on the uninjected side was NF stage 49, the difference in stage compared with the injected side was determined and plotted in the histogram. The hind limb on the injected side was always more advanced, typically by 1 to 1.5 stages (n = 42 injected animals from the same clutch). Tadpoles that lacked a stage difference were not quantified. HL, hind limb.

To visualize the potential phenotypic effects of *TR*α mutations, we injected an mRNA mixture of the two TRα TALEN arms and mCherry into one cell of two cell-stage embryos to disrupt *TR*α on the left or right side of the tadpole body. Embryos were sorted on the third or fourth day (beginning of feeding) into the left side or right side groups based on mCherry expression (Figure 2A). We observed phenotypic differences between the injected and noninjected sides of the body after 2 weeks when hind limb buds appeared at NF stage 46–48 (Figure 2B). The hind limb on the injected side developed faster than one on the noninjected side in 71% (99 of 139 from several injection experiments) of the injected embryos. In the remaining 29%, no difference in the hind limb was observed. When the noninjected side reached NF stage 49, the injected side averaged 1–1.5 NF stages higher (Figure 2D). By the beginning of metamorphic climax 6–8 weeks after injection (NF stage 58–60), most of these F0 animals came to have similar hind limbs on both sides of the body, although some F0 frogs maintained asymmetric hind limbs (data now shown). Stage differences in forelimb development mirrored differences in hind limbs between injected and noninjected sides, but forelimbs on the mutant side emerged from the operculum later than on the noninjected side (data not shown). In addition to limbs, distinctive differences were also found in skin development at later stages of development between noninjected and injected side with a frequency of 20% (7 of 35 surviving tadpoles not used in experiments) (Figure 2C). Specifically, at NF stage 56–57, the nostril, upper jaw, cranium, trunk, hind limb, and forelimb within the operculum on the injected side began to show precocious thicker and more pigmented skin compared with the noninjected side. The skin phenotype frequency was less than for the hind limb because it is less distinctive than standardized morphological limb staging criteria of Nieuwkoop and Faber (35).

### Production of F1 offspring and mutation analysis

**Figure 3.**
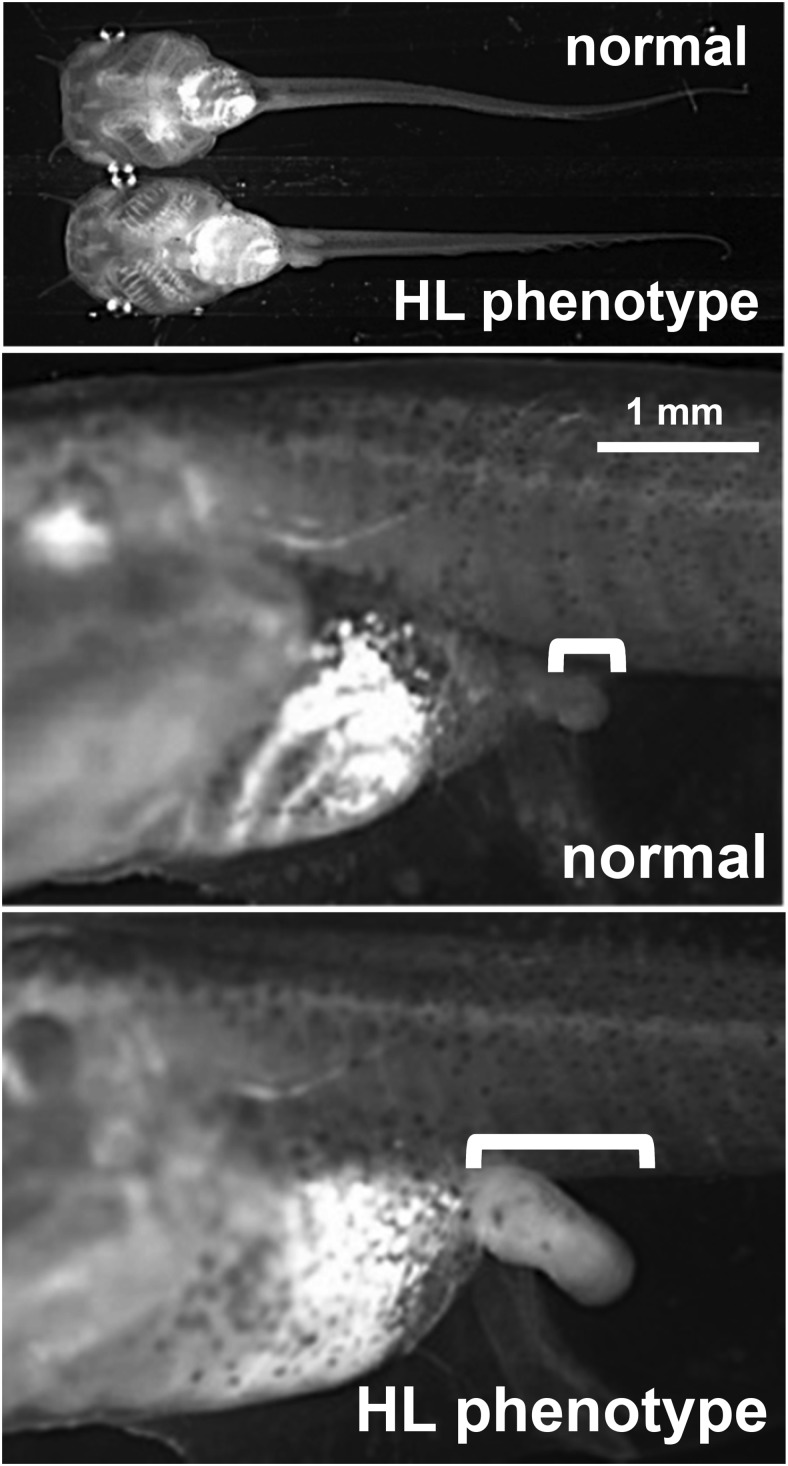
Hind limb phenotype in F1 offspring. Representative sibling offspring from a pair of TRα TALEN founders were imaged at feeding stage (upper panel). The developmental difference in hind limbs is shown in the lower panels. The hind limbs are bracketed. HL, hind limb.

To generate nonmosaic tadpoles with both alleles of *TR*α disrupted, we reared 17 female and 10 male F0 founders that had asymmetric hind limbs as tadpoles. These founders were bred one or two times to each other to obtain a total of 11 clutches (Supplemental Table 1). As expected based on results from the F0 tadpoles, we observed two classes of individuals based on their hind limb phenotype when the tadpoles were 1–2 weeks old, in which some offspring from each of the 11 clutches had more developed fore/hind limbs than expected, given their small body size, when examined near the start of feeding (Figure 3). The frequency of this mutant hind limb phenotype ranged from 2.0% to 40% across all clutches (Supplemental Table 1). The same phenotype was seen in tadpoles reared since feeding (before development of follicles in thyroid gland) in 1 mM methimazole that blocks TH synthesis (data not shown). From three F1 clutches, we sequenced the TRα TALEN target recognition site region from three individuals from each clutch with a normal and mutant phenotype (comprising a total of 79 successful sequencing runs after TOPO cloning) (Supplemental Table 2). We found that in nine of nine cases, individuals with the normal hind limb phenotype had at least one wild-type *TR*α allele, and in nine of nine cases, individuals with the mutant phenotype had two disrupted *TR*α alleles. We found no in-frame mutations in mutant phenotype individuals, but we found one in-frame deletion (six base pairs) in one allele of a normal hind limb individual (Table 1, Supplemental Table 2). All mutations occurred at the TALEN target site between the two zinc fingers of the DNA binding domain, and the frame-shift mutations would abolish the second zinc finger and replace it with 40–50 mutant amino acids followed by an out-of-frame stop codon (Table 1). These results suggest that the mutant phenotype is distinctly recognizable and occurs only upon disruption of both alleles of *TR*α. Also, we detected five to seven *TR*α mutant alleles from a single F0 breeding pair, indicating substantial mosaicism present in the germline of founder animals. Presumably, other tissues in founders were mosaic, justifying the need to produce F1 offspring for confirmation of the mutant phenotype and for further analysis of the effects of the *TR*α mutation.

**Table 1.** Amino Acid Sequence Alignment at TRα TALEN Target Site

Cl	Ph	Ind	Al	Amino Acid Sequence	Length
1	Normal	1	1, 2	IQKNLHPSYSCKYDGCCI	312
1	Normal	2	1	IQKNLHPSYSCKYDGCCI	312
1	Normal	2	2	IQKNLHPS--CKYDGCCI	312
1	Normal	3	1	IQKNLHPSYSCKYDGCCI	312
1	Normal	3	2	IQKNLHPS**FMQVRWLLHY**	46
1	HL	1	1	IQKNLHPSY**HASTMAAAL**	51
1	HL	1	2	IQKNLHPSY**RASTMAAAL**	51
1	HL	2	1	IQKNLHPSY**MQVRWLLHY**	46
1	HL	2	2	IQKNLH**LHMQVRWLLHYR**	45
1	HL	3	1	IQKNLHPS**FHASTMAAAL**	51
1	HL	3	2	IQKNLHPS**FMQVRWLLHY**	46
2	Normal	1	1	IQKNLHPSYSCKYDGCCI	312
2	Normal	1	2	IQKNL**QVRWLLHYRQDHP**	41
2	Normal	2	1	IQKNLHPSYSCKYDGCCI	312
2	Normal	2	2	IQKNLHPS**IMQVRWLLHY**	46
2	Normal	3	1	IQKNLHPSYSCKYDGCCI	312
2	Normal	3	2	IQKNL**QVRWLLHYRQDHP**	41
2	HL	1	1	IQKNLHPS**FMQVRWLLHY**	46
2	HL	1	2	IQKNLHPS**YIHASTMAAA**	52
2	HL	2	1	IQKNLHPS**FHASTMAAAL**	51
2	HL	2	2	IQKNL**QVRWLLHYRQDHP**	41
2	HL	3	1	IQKNLH**DQHASTMAAALS**	50
2	HL	3	2	IQKNL**QVRWLLHYRQDHP**	41
10	Normal	1	1	IQKNLHPSYSCKYDGCCI	312
10	Normal	1	2	IQKNLHPSY**YASTMAAAL**	51
10	Normal	2	1	IQKNLHPSYSCKYDGCCI	312
10	Normal	2	2	IQKNLHPS**HASTMAAALS**	50
10	Normal	3	1	IQKNLHPSYSCKYDGCCI	312
10	Normal	3	2	IQKNLHPSY**HASTMAAAL**	51
10	HL	1	1	IQKNLHPSY**YASTMAAAL**	51
10	HL	1	2	IQKNLHPSY**HASTMAAAL**	51
10	HL	2	1	IQKNLHPS**HASTMAAALS**	50
10	HL	2	2	IQKNLHPSY**HASTMAAAL**	51
10	HL	3	1, 2	IQKNLHP**SFHASTMAAAL**	51

Abbreviations: Cl, clutch identity; HL, hind limb; Ind, individual; Ph, normal or hind limb phenotype; Al, allele (in two individuals either one allele was recovered or both alleles were the same sequence). Amino acid sequence in bold are mutant amino acids due to frame-shift mutations, and dashes are deleted amino acids due to in-frame mutations. Length is the number of amino acids in which 312 is the remaining wild-type sequence and 45–52 is the number of mutant amino acids C terminal to those shown.

### The absence of TRα releases gene repression and decreases tissue responsivity

**Figure 4.**
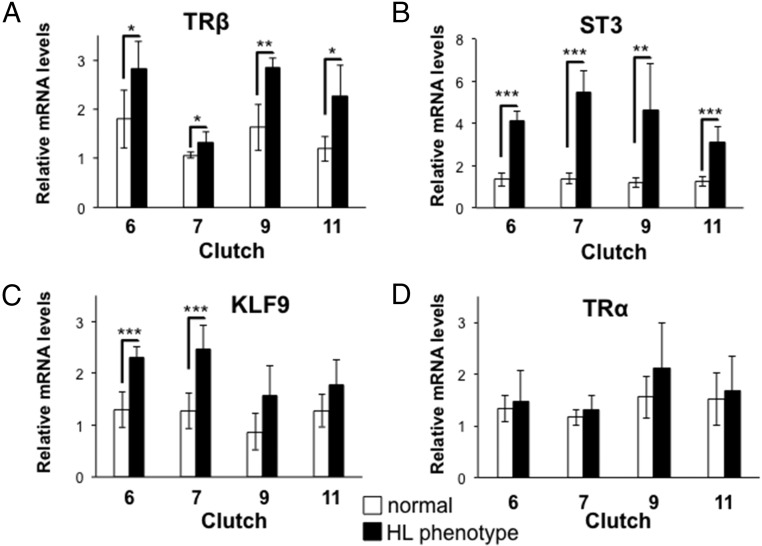
Derepression of TH-response genes in hind limb phenotype tadpoles. Total RNA from whole bodies of F1 normal or hind limb (HL) phenotype tadpoles at NF stage 48–49 and NF stage 50–51, respectively, was isolated from four clutches [clutches 6, 7, 9, and 11 (Supplemental Table 1)] was isolated to analyze mRNA expression before TH circulation begins at NF stages 54–55. HL phenotype animals had higher expression levels of *TR*β (A), *ST3* (B), and *KLF9* (C) mRNA than normal animals. D, The TALEN-induced mutation in *TR*α had no effect on its mRNA expression levels, which were not significantly different in normal and hind limb phenotype animals (n = 4–6); bars show expression levels relative to the housekeeping gene *rpL8*, and error bars represent SD. Significance levels for a one-way ANOVA were: *, *P* < .05; **, *P* < 0.01; ***, *P* < 0.001.

The dual-function model hypothesizes that the lack of TRα would derepress TH-response genes in premetamorphosis in the absence of T_3_. We measured the mRNA levels of three TH-response genes, *TR*β, *ST3*, and *KLF9*, in sibling normal or mutant phenotype tadpoles. Tadpoles from four clutches were collected, in which mutants had hind limbs at NF stage 50–51 and normal tadpoles had hind limbs at NF stage 48–49 and all had similar snout vent length as in Figure 3. Mutant phenotype tadpoles expressed higher levels of *TR*β, *ST3*, and *KLF9* mRNA than normal phenotype tadpoles in the absence of exogenous TH (Figure 4, A–C). We also measured TRα mRNA levels using primers to exons in the ligand binding domain and found no difference between normal and mutant phenotype individuals in TRα mRNA levels (Figure 4D).

**Figure 5.**
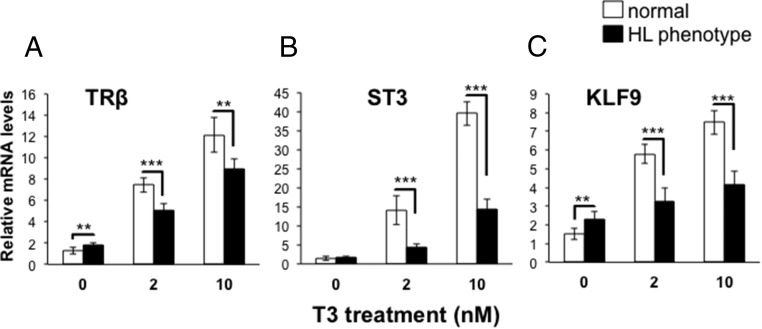
Impaired induction of TH-response genes in hind limb phenotype tadpoles. Total RNA from whole bodies of sibling F1 normal or hind limb (HL) phenotype tadpoles at NF stages 48–49 and NF stages 50–51, respectively, was isolated after treatment with 0, 2, or 10 nM T_3_ for 24 hours. The mRNA expression levels of *TR*β (A), *ST3* (B), and *KLF9* (C) were significantly higher in normal tadpoles treated with 2 or 10 nM T_3_. Uninduced levels were increased for *TR*β and *KLF9* in HL phenotype tadpoles, as in Figure 4. n = 4–6, bars show expression levels relative to the housekeeping gene rpL8, and error bars represent standard deviation. One-way ANOVA showed significant differences for most comparisons, *P* < .05; *, < 0.01; **, < 0.001; ***.

In addition to the role for unliganded TR predicted by the dual-function model, another role is to render tissues sensitive and/or more responsive to the TH signal. To address this issue, we measured gene expression and observed morphological changes in sibling normal and hind limb phenotype tadpoles at NF stage 48–49 and 50–51, respectively, treated with exogenous T_3_. As predicted, levels of *TR*β, *ST3*, and *KLF9* mRNA were higher in normal compared with hind limb phenotype animals after treatment with exogenous T_3_ at both concentrations used (2 and 10 nM) (Figure 5). Interestingly, T_3_ increased the expression levels of these TH-response genes 2-fold or more in hind limb phenotype animals compared with the 0 nM T_3_ treatment, suggesting functional TRβ in early larval stages. However, given that we did not sequence verify every individual, we cannot rule out residual TRα activity from a hypomorphic allele.

**Figure 6.**
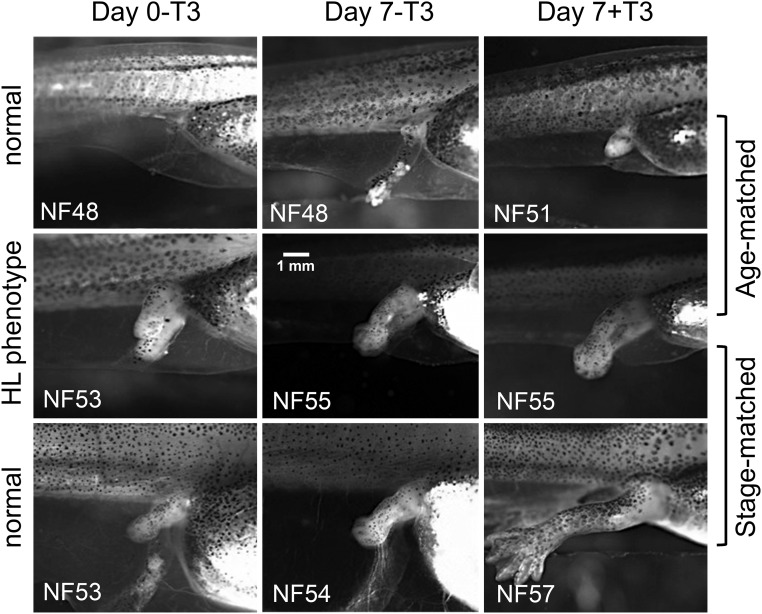
Reduced hind limb responsivity to TH in hind limb phenotype tadpoles. Normal and hind limb (HL) mutant phenotype tadpoles from the same clutch were treated with 0 or 10 nM T_3_ for 7 days. Hind limbs in mutant phenotype tadpoles (middle row) were compared with normal tadpoles of the same age (top row, age matched) or at the same stage (bottom row, stage matched). In the absence of T_3_, hind limbs advanced up to two stages in 7 days in normal and mutant phenotype tadpoles, (compare day 0-T_3_ and day 7-T_3_). In the presence of T_3_ (compare day 0-T_3_ and day 7+T_3_), hind limbs in normal tadpoles advanced from NF stage 48 to NF stage 51 (top row) or from NF stage 53 to NF stage 57 (bottom row), whereas hind limbs of mutant phenotype tadpoles advanced from NF stages 53 to NF stage 55. Importantly, the effect of T_3_ in mutant hind limbs (no difference in stage but slight increase in hind limb length) was greatly reduced compared with the effect in normal tadpoles of the same stage (compare day 7-T_3_ and day 7+T_3_). Hind limb images were photographed with the same magnification. This experiment was repeated with a sample size of four to six with similar results.

**Figure 7.**
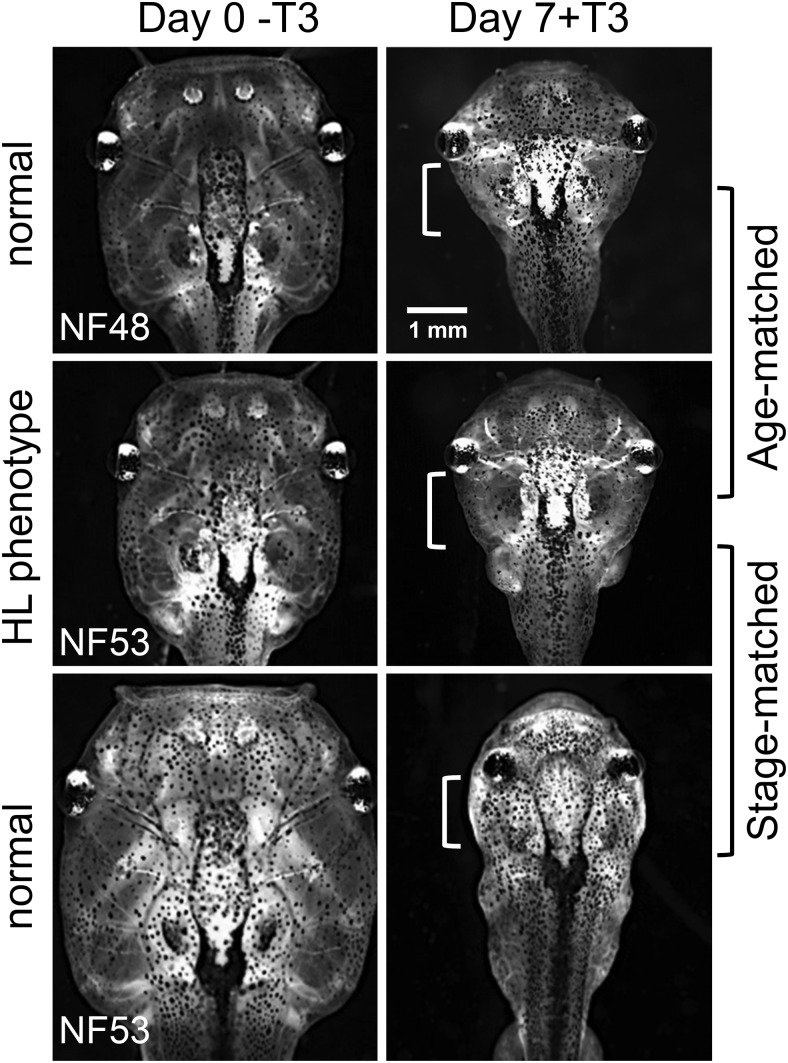
Reduced gill responsivity to TH in hind limb phenotype tadpoles. Normal and hind limb (HL) mutant phenotype tadpoles were the same individuals as in Figure 6. Gills in mutant phenotype tadpoles (middle row) were compared with normal tadpoles of the same age (top row, age matched) or at the same stage (bottom row, stage matched). In the presence of T_3_, gill resorption in normal tadpoles (top and bottom rows) occurred to a greater extent than in mutant phenotype tadpoles (middle row) (the clear area seen in mutant phenotype tadpoles is larger than in normal tadpoles, near white brackets). Gill images were photographed with the same magnification. This experiment was repeated with a sample size of four to six with similar results.

To compare tissue sensitivity/responsivity at the morphological level, we compared responses of hind limb and gill in normal and mutant phenotype sibling tadpoles at the same size and age (age matched) as well as with larger, older normal tadpoles of the same hind limb stage (stage matched) after 7 days of exogenous T_3_ treatment. Hind limbs of the age-matched normal tadpoles advanced in development from NF stage 48 to NF stage 51 in the presence of T_3_ but not in the absence of T_3_ (Figure 6, top row). Hind limbs of the stage-matched normal tadpoles advanced in development from NF stage 53 to NF stage 54 in the absence of T_3_ and to NF stage 57 in the presence of T_3_ (Figure 6, bottom row). Hind limbs in the mutant phenotype tadpoles advanced from NF stage 53 to NF stage 55 without exogenous T_3_, which was one stage higher than the stage-matched control, and exhibited very little response to T_3_, ie, the length of the hind limb increased slightly due to T_3_ treatment but toe differentiation was not induced (Figure 6, middle row). For the gills, 7 days of treatment with 10 nM T_3_ caused complete gill resorption in age-matched and stage-matched normal tadpoles at NF stage 48 and NF stage 53, but the process was not quite complete in the mutant phenotype tadpoles (Figure 7). In the absence of T_3_, gill morphology did not change during the 7-day treatment period in normal or mutant phenotype tadpoles (data not shown).

## Discussion

Two nonmutually exclusive roles of unliganded TRα in the absence of TH during premetamorphosis have been proposed: 1) repress TH target genes required to initiate metamorphosis and 2) regulate tissue sensitivity/responsivity to TH underlying the timing of developmental events. Here we used TALEN gene disruption technology to disrupt *TR*α and provide direct evidence for both of these roles in frog development. We found higher expression of TH-response genes in the absence of TH, decreased responsivity to induction by TH, and precocious initiation of developmental progression in tadpoles with both *TR*α alleles disrupted.

We targeted a region in between the two zinc fingers in the DNA binding domain of TRα, and the disruption was sufficiently efficient to produce a high frequency of tadpoles with mutant phenotypes in the injected F0 individuals. However, the range in degree of hind limb phenotypes in founder animals suggested mosaic gene disruption among cells and/or production of mutant TRα proteins of altered function rather than null mutations. The 11 clutches of F1 offspring from paired founders exhibited variable frequencies of germline transmission (2.0%–40%), suggesting mosaicism among germ cells in gonads of founder animals. Nevertheless, a consistent hind limb phenotype within F1 offspring (discussed in more detail below) was observed. This mutant phenotype perfectly coincided with frame-shift mutations between the two zinc fingers and subsequent mutant amino acids and out-of-frame stop codon, which are not known to leave TRα with any residual activity. Mutations in the second zinc finger abrogate effects on transcription (39) and heterodimerization is required for efficient binding to DNA (40). The truncations in our study eliminate the second zinc finger and the dimerization domains, suggesting complete loss of DNA binding and gene regulation. In mammals, TRα1 is the bona fide receptor, with TRα2 and TRα3 as the known splice variants (41), TRα2 and TRα3 do not bind TH and may act as weak dominant negatives. Such variants, if present in frogs, would be disrupted in our tadpoles. Also, in mammals, TRaΔ1 and TRaΔ2 are generated from an internal promoter with a transcription start site at an exon 3′ of our TALEN-induced mutations and cannot bind DNA or TH and have limited tissue distribution (42). These isoforms, if present in frogs, would not be expected to have a significant impact on external morphology or whole-body measurements of gene expression and thus would not alter the conclusions here about TRα-mediated gene regulation. We cannot rule out off-target effects of the TRα TALENs or some transactivation activity of the A/B domain of TRα that would remain intact, but we note that the phenotypes observed to date fully comply with expectations regarding *TR*α mutations.

TH induces metamorphosis by up-regulating TH-response genes, including *TR*β, *ST3*, and *KLF9* measured herein (18, 20, 44). The dual-function model predicts that these same genes will be repressed by TR in the absence of TH to delay metamorphosis until endogenous TH reaches circulation (10). We found increased expression of these genes in mutant phenotype animals in the absence of TH before metamorphosis. At the level of morphology, precocious limb development in early larvae is consistent with derepressed levels of TH-response genes responsible for metamorphic initiation. The same phenotype after methimazole treatment rules out the possibility that *TR*α disruption caused a precocious increase in TH production to explain the increase in TH-response gene expression and precocious hind limb development. Thus, our data suggest that TRα represses TH-response genes during premetamorphosis to delay hind limb development prior to TH in circulation. In addition to derepression, mutant phenotype animals revealed the second predicted role for unliganded TRα, decreased sensitivity/responsivity to TH. In the absence of TRα, gene induction by TH is expected to be absent or weak due to the low TRβ expression levels in premetamorphic tadpoles. As predicted, the expression levels of *TR*β, *ST3*, and *KLF9* in mutant phenotype tadpoles were significantly lower than those in normal tadpoles treated with exogenous T_3_, indicating impaired gene induction in the mutant phenotype tadpoles.

Even though all tissues of the tadpole require TH for transformation, we observed precocious development only in the hind limb in mutant phenotype tadpoles. In general, hind limb buds come out at an early stage (NF stage 46) and grow and develop slowly until NF stage 54 when hind limb growth becomes dependent on TH and the tadpole has increased its body size 3-fold. Hind limbs are known to express high levels of TRα early (NF stage 52), compared with other organs (28, 46). In addition, TRβ is detectable at later stages in the hind limb by immunocytochemistry, restricted to regions of cartilage-forming cells (47). Thus, it is consistent that the absence of TRα would be first and best revealed in the hind limb. The gills retained to a great extent the ability to resorb after T_3_ treatment in mutant phenotype tadpoles, indicating TRα may not play as dominant a role in gill compared with hind limbs. Indeed, the TRβ-selective agonist GC-1 caused complete gill resorption but less hind limb growth and elongation compared with T_3_, whereas a TRα-selective agonist CO23 induced hind limb growth preferentially over gill resorption (43, 45). The remaining low responsivity to TH in hind limb and substantial responsivity in gill in the absence of TRα is most likely due to premetamorphic TRβ expression and/or nongenomic actions of TH.

The unliganded function of TR has been shown in the cerebellum, heart, and under hypothyroid conditions in the cochlea in mammals (5–7). As in tadpoles, the expression of TR before the onset of thyroid gland in mammalian fetuses has engendered the idea that TR functions in the unliganded state. Although TH may pass through the placenta, it is unlikely that all or most TRs in the fetus are occupied by TH (1). The lack of known widespread actions of unliganded TR in mammals may be because low levels of TH are present in the fetus such that effects of unliganded TRα may appear only in hypothyroid conditions as in the cochlea. Because frogs exist for a long period of time in the absence of TH (3 wk of premetamorphosis), TH-dependent development in TRα-disrupted frogs may be a good model to reveal the effects of unliganded TR difficult to uncover in the mammalian model.
